# Clitoral Abscess: A Case Report Highlighting an Uncommon Source of Gynecological Emergency

**DOI:** 10.7759/cureus.50114

**Published:** 2023-12-07

**Authors:** Mohamed Ferhi, Nadia Marwen, Chourouk Alouen

**Affiliations:** 1 Psychiatry, Ibn El Jazzar University Hospital, Kairouan, TUN; 2 Obstetrics and Gynaecology, Ibn El Jazzar University Hospital, Kairouan, TUN

**Keywords:** clitoral abscess, emergency obstetrics, perineal abscess, clitoris, minimally invasive gynecologic surgery

## Abstract

Clitoral abscess is a rare gynecological condition with limited literature reports. This case report aimed to contribute to the understanding and management of clitoral abscesses, particularly in cases without identifiable causes. A 26-year-old sexually active woman with no significant medical history presented with severe perineal pain originating specifically in the clitoral region. The onset of symptoms, characterized by pulsatile perineal pain worsening over several days, was localized to the clitoris. Clinical examination indicated a clitoral abscess, confirmed through surgical incision and drainage. The medical treatment consisted of the administration of oral analgesics, flucloxacillin at a dose of 500 mg for seven days, and non-steroidal anti-inflammatory agents. The procedure preserved the dorsal artery of the clitoris, with bacteriological analysis revealing polymicrobial organisms. Post surgery, the patient recovered fully with no complications. By sharing details about this unusual clinical case and its effective treatment, we contribute to advancing the collective knowledge in the field. This dissemination supports a deeper understanding and facilitates informed decision-making for healthcare professionals.

## Introduction

Clitoral abscess, an infrequently encountered gynecological condition, remains a relatively enigmatic entity with approximately 17 reported cases in the literature [1,2]. While certain cases of this condition can be attributed to recognizable factors like pilonidal disease or genital trauma resulting from practices such as female circumcision or genital mutilation, there are instances where it occurs without an identifiable cause [2]. Clitoral abscess manifests as a localized, painful, and fluctuant inflammatory lesion enveloping the clitoris. It can elicit substantial morbidity, inducing severe vulvar pain, dysuria, vulvar swelling, and erythema of the clitoral hood in women of reproductive age. In premenarchal girls, this condition assumes particular significance, given its potential to exert deleterious effects on future reproductive health [3,4]. Management typically involves a comprehensive strategy that includes antibiotic therapy, alongside surgical procedures, particularly incision and drainage accompanied by marsupialization [1,2]. Unfortunately, the effectiveness of these interventions in preventing a relapse remains limited. In 2012, Koussidis [2] conducted a literature review on this pathology, reporting 17 cases, which did not provide guidelines on the subject or outcomes of bacteriological cultures. Therefore, the available treatment options are primarily founded on individual clinical experience rather than a sufficient body of available evidence. In summary, our case report provides valuable insights into clitoral abscess management. By sharing this rare clinical presentation and successful intervention, we contribute to the collective knowledge in the field, fostering better understanding and informed decision-making among healthcare practitioners.

## Case presentation

**Figure 1:**
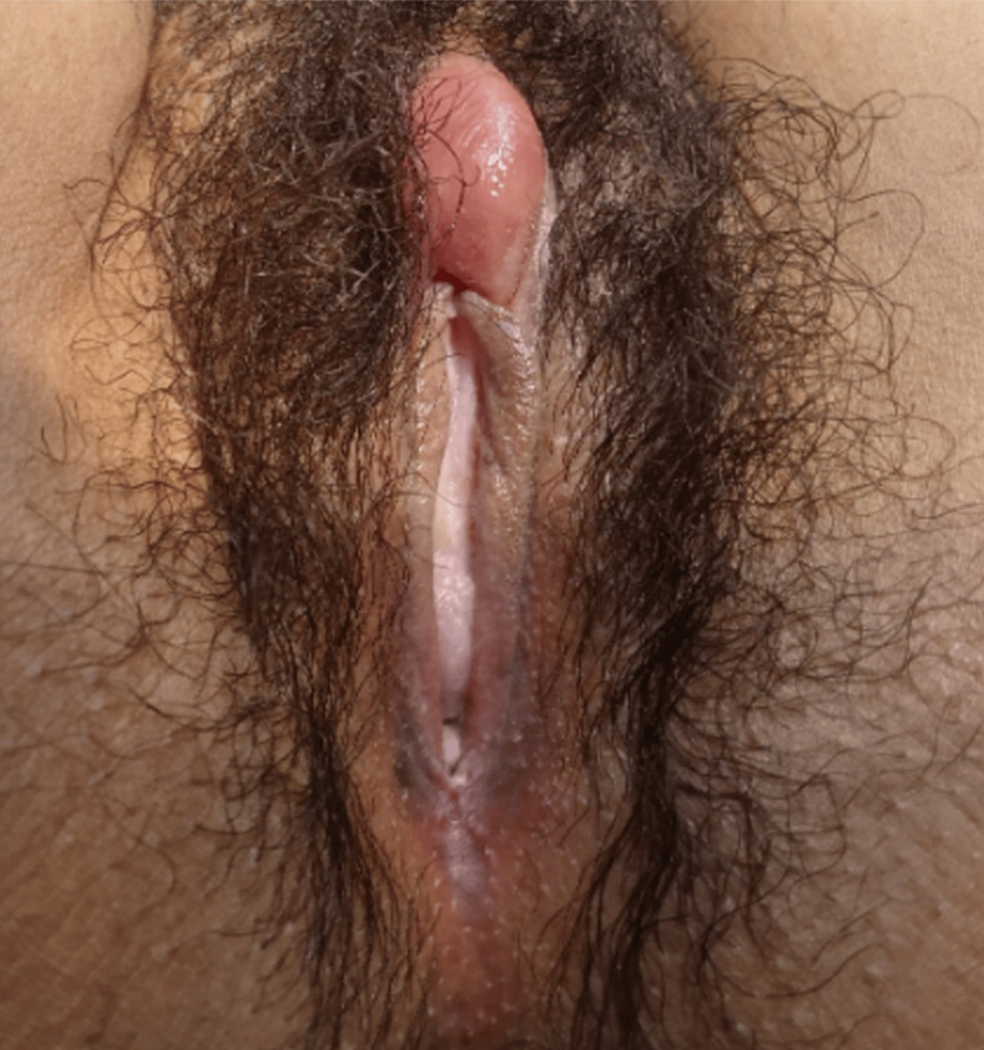
The clitoral abscess

A 26-year-old woman, nulligravida and sexually active, was referred to the emergency department of gynecology in Ibn El Jazzar University Hospital in March 2023, complaining of perineal pain. Her medical history revealed a history of smoking, with no other significant medical conditions or ongoing medications. Notably, she was not immunocompromised. The patient reported no sexual history, including male or female sexual encounters, no use of sexual "toys," and no unusual sexual habits. The patient did not report any history of vaginal discharge or prior vulval pruritus during the clinical evaluation. The onset of her symptoms was characterized by pulsatile perineal pain, worsening over the days. Importantly, the patient did not exhibit any signs of fever. Four days following the initial onset of symptoms, both the pain and localized swelling experienced a significant escalation, culminating in a pain score of 9 out of 10. The clinical examination unveiled a tense, spherical clitoral mass upon palpation, accompanied by localized inflammatory indicators. There was no evidence of skin breakdown or necrosis. The remainder of the vulvar and cutaneous examination did not reveal any abnormalities (Figure 1). 

Imaging was considered unnecessary in this context as it concerned a localized infection and no signs of adenopathy were observed. The diagnosis of clitoral trauma and swelling was ruled out as the patient reported no sexual activity for one week before the onset of symptoms and there was no history of activities causing genital discomfort. Furthermore, no signs of candidal infection or irritation were observed. Consequently, the diagnosis of a clitoral abscess was confirmed.

The surgical procedure was undertaken, involving incision and drainage of the abscess. Additionally, a biopsy of the abscess was performed and sent for histopathological analysis. The results indicated inflammatory changes characterized by neutrophilic infiltrates, fibrinoid necrosis, and granulation tissue formation, consistent with an abscess. No evidence of malignancy was identified upon microscopic examination. During the surgical intervention, particular care was taken to preserve the integrity of the dorsal artery of the clitoris to prevent any inadvertent damage. Approximately 150 cc of pus was extruded from the abscess. A bacteriological sample was sent to the laboratory, and subsequent analysis revealed the presence of polymicrobial organisms, without any specific bacterial identification.

Immediately following the surgical procedure, clitoral tension had completely regressed, and the patient no longer reported any discomfort or pain. The medical treatment consisted of the administration of oral analgesics, flucloxacillin at a dose of 500 mg for seven days, and non-steroidal anti-inflammatory agents. A few days after the initial treatment, a notable improvement in the patient's symptoms was observed, with a significant reduction in both the swelling and discomfort. One month later, upon reevaluation, the patient had completely recovered, experiencing neither pain nor any neurological sequelae related to the clitoral hood. The clitoral hood had healed with a fully restored anatomical appearance, without any scar or aesthetic concerns.

## Discussion

Clitoral abscess, a limitedly reported gynecological condition, can manifest either spontaneously or as a result of identifiable causes such as pilonidal disease or genital trauma. The condition presents as a localized, painful inflammatory lesion surrounding the clitoris, causing significant morbidity. In our case, the patient was 26 years old, which falls within the typical age range for individuals affected by clitoral abscess, typically between 20 and 30 years [4-10]. Reviewing the literature revealed cases with onset ages ranging from 8 to 53 years [1,11].

While no specific risk factors have been discerned, some case reports have mentioned active smoking [1] and pregnancy [4] as potential associations with clitoral abscesses. Evidence suggests that sexually transmitted diseases do not appear to be causative factors for periclitoral abscess [1,2]. Our case also had no history of sexually transmitted diseases. In certain cases, clitoral abscesses can progress to necrotizing fasciitis, especially in elderly patients with underlying comorbidities like immunocompromised states, diabetes, or prolonged corticosteroid therapy [12].

The majority of clitoral abscess cases lack an identifiable cause, which was the case in our study. However, some cases have been reported as complications of pre-existing pilonidal sinus tracts of the clitoris [1,5-7,9,10], Crohn's disease with extension to the vulva [8], or ectopic breast tissue [13]. Polymicrobial infections are the predominant cause in most instances which aligns with the microbiological results of this present case. Some studies reported *Staphylococcus aureus* [2,4,8], *Staphylococcus epidermidis*, *Peptostreptococcus* [8], *Streptococcus bovis* [14], *Bacteroides*, and diphtheroids [4]. 

There is no established optimal management for clitoral abscesses, and available treatment options are primarily based on individual clinical experience rather than a robust body of evidence. Some cases reported spontaneous drainage [3,4,8], while others required excision of the cyst track in the case of infected pilonidal cysts [5-7,9-11]. In many instances, incision and different antibiotic therapies, including sulfadiazine [5], ciprofloxacin [15], cefazolin+metronidazole [10], and amoxicillin-clavulanic acid [1], were employed for treating the initial episode. However, no particular treatment approach emerged as superior. In cases of recurrent episodes, marsupialization was performed with promising results and no reported recurrence, aligning with findings in the literature [4,14]. The recurrent episodes and the success of marsupialization warrant further investigation to establish the most effective management strategy for clitoral abscess.

## Conclusions

The lack of comprehensive guidelines and bacteriological culture outcomes poses challenges. Our case study, a 26-year-old woman with no identifiable risk factors, highlights the need for further research to establish practical treatment approaches and preventive measures for clitoral abscesses. This case underscores the importance of expanding our understanding of this condition to improve clinical outcomes.
